# Flash Communication: A Dithiolene-Based Hexathiadisila[4.4.4]propellene

**DOI:** 10.1021/acs.organomet.6c00227

**Published:** 2026-08-12

**Authors:** John C. Johnson, Yuzhong Wang, Claire Buck, Pingrong Wei, Mitchell E. Lahm, Henry F. Schaefer, Gregory H. Robinson

**Affiliations:** Department of Chemistry and the Center for Computational Chemistry, 1355 The University of Georgia, Athens, Georgia 30602-2556, United States

## Abstract

Room-temperature reaction of the imidazole-based dithiolate dimer (**1**) with hexachlorodisilane (Cl_3_Si-SiCl_3_) in toluene affords **2**—a chiral hexathiadisila[4.4.4]­propellene with a silicon–silicon bridgehead bond [d_Si–Si_ = 2.2947(19) Å, av]. Compound **2** is the first dithiolene-based unsaturated propellane. X-ray structural analysis confirms that **2** exists in P- and M-helical forms in the solid state. Density functional theory (DFT) computations reveal that the helical chirality of **2** originates from steric interactions between the bulky substituents of the dithiolene ligands.

## Graphical abstract

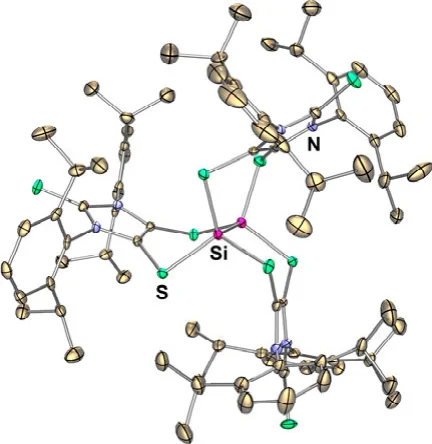

Propellanes constitute an unusual class of tricyclic molecules wherein three rings are fused through a bond between two bridgehead central atoms.^1–3^ [1.1.1]­Propellane (**A** in Figure 1, n = 1) serves as the propellane archetype. Propellanes are intriguing due to not only their unique bonding properties and interesting chemical reactivity but also their applications in organic synthesis,^4^ pharmaceutical research,^3–5^ and material sciences (such as synthesis of rigid-rod polymers).^3^ Since the term “propellane” was coined by Ginsburg six decades ago,^6^ the chemistry of these interesting molecules has been extensively explored.^3^ Although 1,6-disila[4.4.4]­propellane (**B** in Figure 1)—the first propellane containing a central Si–Si bond—was reported in 1971,^7–9^ reports of such propellanes remain rare. Pentasila[1.1.1]­propellane (**C** in Figure 1) and 3,7,10-trichalcogenaoctasila[3.3.3]­propellanes (**D** in Figure 1) are two representative examples.^10,11^ Notably, dithiolene-based propellenes with a central Si–Si bond (**E** in Figure 1) have not been reported.

**Figure 1:**
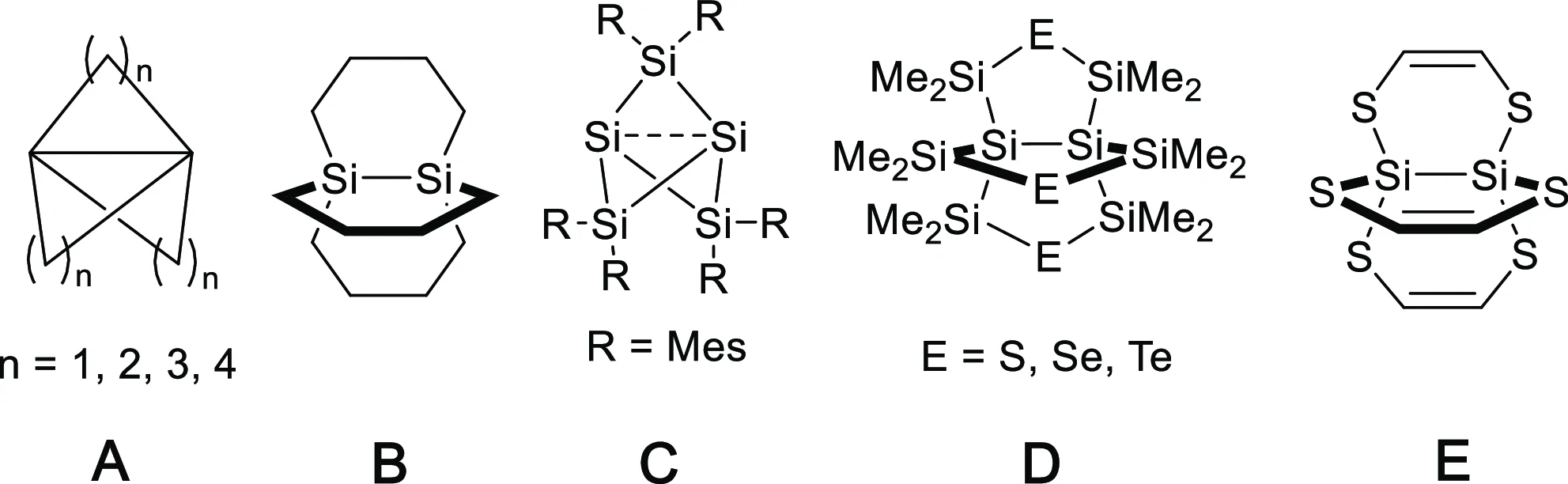
Propellanes (**A**); propellanes bearing a central Si–Si bond (**B**–**D**); the “hexathiadisila[4.4.4]­propellene” core of **2** (**E**).

Due to the redox noninnocent properties of the dithiolene ligand,^12^ transition-metal dithiolene complexes have demonstrated wide capabilities in materials science (such as optoelectronic devices)^13,14^ and biological systems (i.e., molybdenum and tungsten enzymes).^15,16^ However, the chemistry of main group element-based dithiolene complexes has not developed in parallel with that of their transition-metal counterparts. For example, silicon­(IV)-based dithiolene complexes have long been dominated by four- and five-coordinate bis­(dithiolene) complexes (Figure 2 **A,B**).^17–19^ The corresponding six-coordinate tris­(dithiolene) complex (Figure 2 **C**) was not reported until 2020.^20^ The dithiolate derivative of disilane (Figure 2 **D**) represents the only binuclear silicon­(III) bis­(dithiolene) complex containing a silicon–silicon bond.^17^ Recently, a series of five-coordinate amidinate- and dithiolene-based Si­(II) and Si­(IV) complexes were also reported.^21,22^

**Figure 2:**
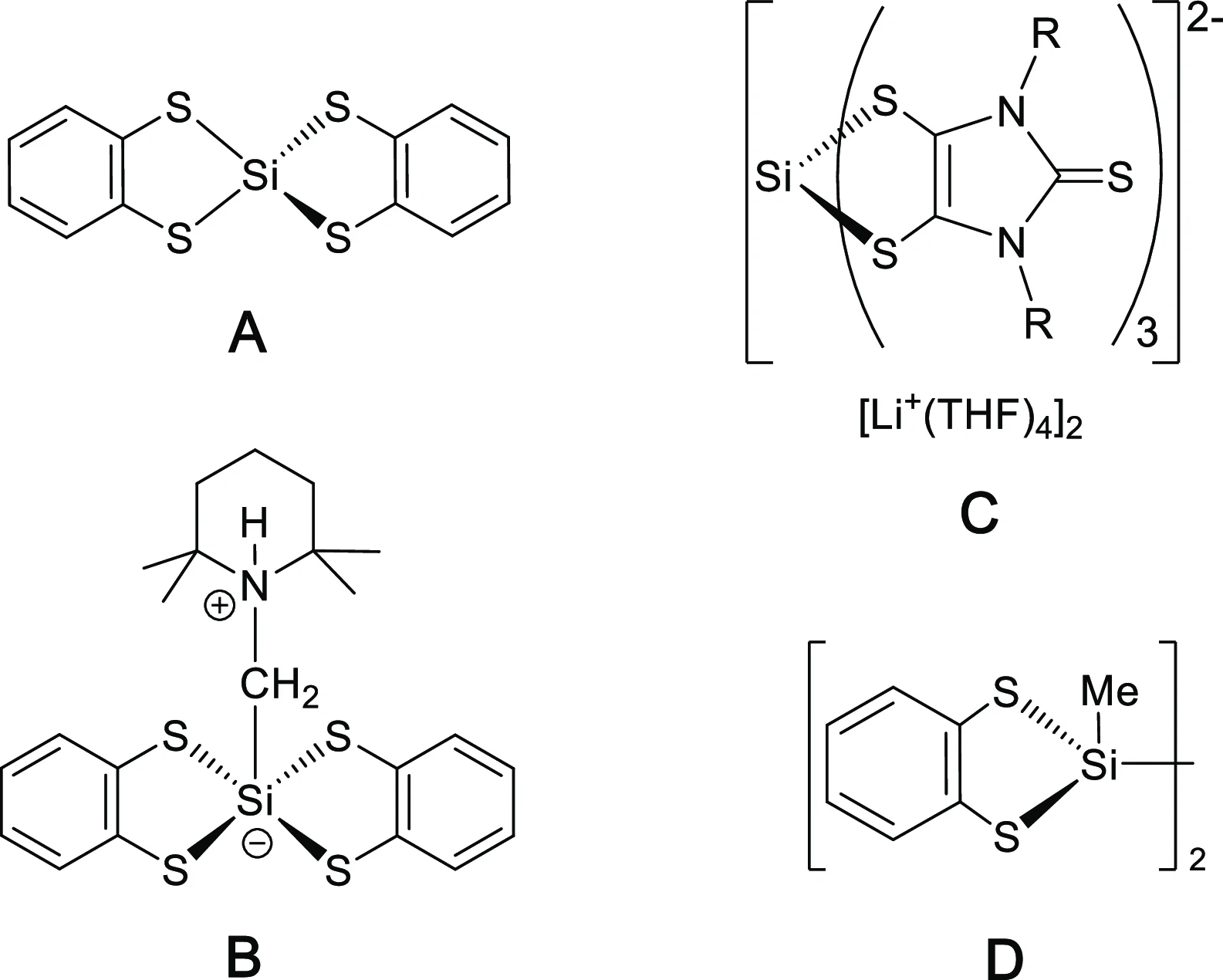
Mononuclear (**A**–**C**) and binuclear (**D**) silicon-based dithiolene complexes.

It should be noted that all the dithiolene ligands in these reported silicon-dithiolene complexes (**A**–**D** in Figure 2) act as a bidentate ligand. Herein, we report the synthesis,^23^ structure,^23^ and computations^23^ of a chiral hexathiadisila[4.4.4]­propellene with a central silicon–silicon bond (**2**). Notably, **2** represents the first report of a dithiolene-based unsaturated (i.e., containing C=C double bonds) propellane.

This laboratory synthesized the first stable lithium-based dithiolene radical by trisulfurization of an anionic N-heterocyclic dicarbene,^24^ which was readily converted to the corresponding dithiolate dimer (**1**) via potassium graphite reduction.^25^ Room-temperature reaction of **1** with (*light-sensitive*) hexachlorodisilane (in a 3:2 molar ratio) in toluene—protected from light—gives **2** as pale-yellow crystalline solid (in 65% yield) (Scheme 1). X-ray-quality single crystals of **2** (colorless) were obtained by recrystallization in a toluene/hexanes solvent mixture. Notably, the **2′** isomer, containing one bridging and two bidentate dithiolene ligands (Scheme 1), was not obtained. DFT computations^23^ (at the B3LYP/6-311G** level) show that the simplified **2-Me** model is 8.67 kcal·mol^–1^ lower in energy than the **2′-Me** model, supporting the experimental observation. The rigid conjugated imidazole-based dithiolene ligand may incur greater distortion upon chelation and thus favor a bridging mode across the Si–Si unit. While being thermally stable (**2** melts and decomposes gradually above 275 °C), **2** is highly moisture- and oxygen-sensitive. The ^29^Si­{^1^H} NMR resonance of **2** (14.6 ppm) shifts downfield compared to that (−28.9 ppm) of 1,6-disila[4.4.4]­propellane (Figure 1 **B**).^9^

**Scheme 1:**
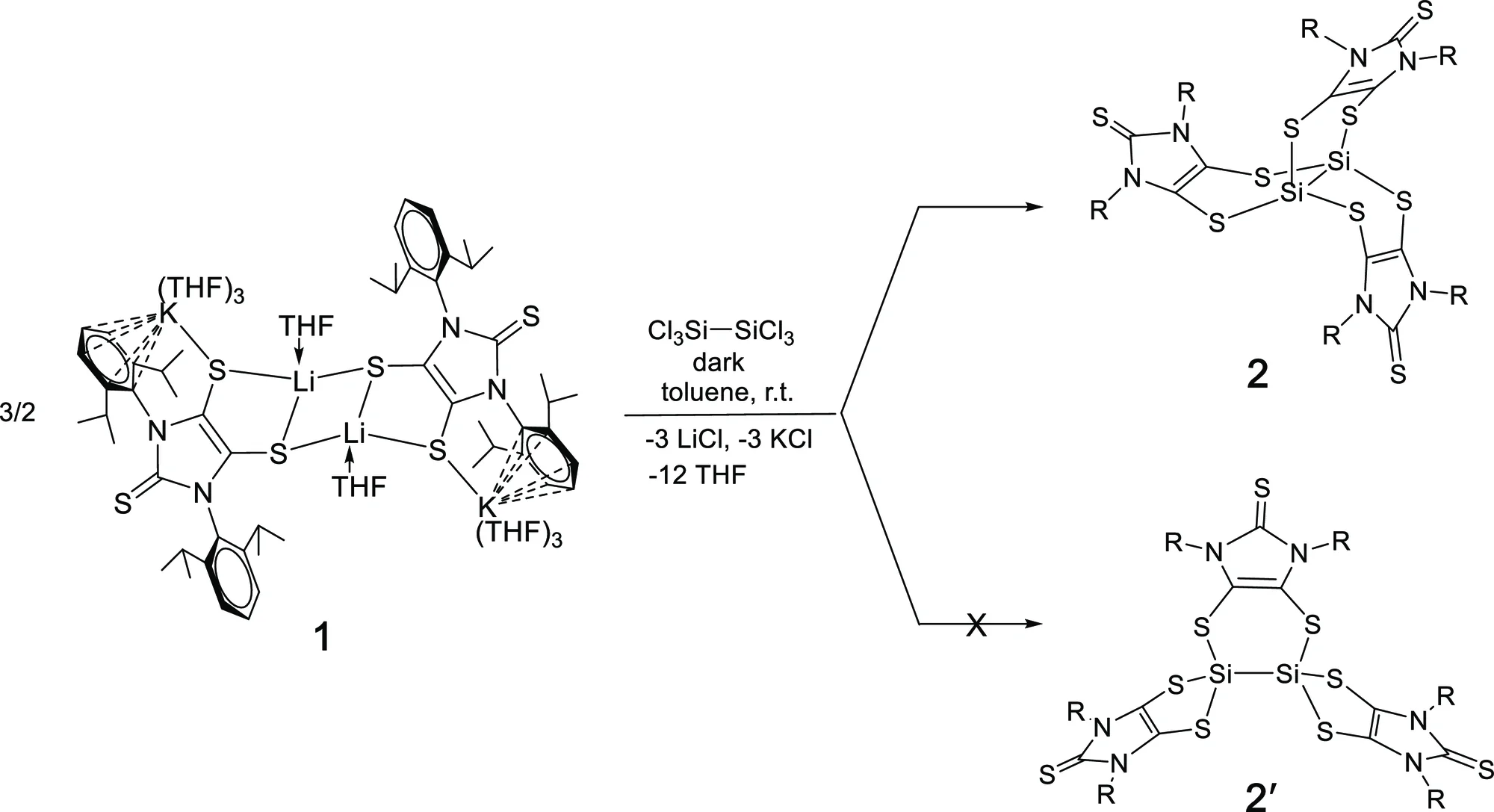
Synthesis of **2** (R = 2,6-Diisopropylphenyl)

The asymmetric unit of the crystals of **2** contains one pair of P- and M-helical enantiomers (the helical descriptors P and M are used to describe the chirality of **2**, Figure 3a,b).^23^ For clarity, only the selected bonding parameters of **2**-P are shown in the caption of Figure 3. Compound **2** possesses approximate *C*_3_-symmetric propeller geometry. According to DFT computations (at the B3LYP/6-311G** level),^23^ the simplified **2-Me** model has *C*_3*h*_-symmetric propeller geometry (with a S–Si–Si–S torsion angle of 0°) (Figure 3c) and is therefore achiral. Sterically demanding substituents (i.e., 2,6-diisopropylphenyl) of the dithiolene ligands in **2** enforce unidirectional twisting of the three C_2_S_2_Si_2_ lobes around the silicon–silicon bridgehead axis [S–Si–Si–S torsion angles: 18.4° (av) for **2**-P; −17.4° (av) for **2**-M], lowering the symmetry to approximately *C*_3_. Consequently, **2** exhibits helical chirality. In addition, the three six-membered C_2_S_2_Si_2_ rings in **2** are bent along the S···S axis in the same direction. While being somewhat shorter than that of the simplified **2-Me** model (2.337 Å),^23^ the central Si–Si bond of **2** [2.2947(19) Å, av] compares well to that of the disilapropellane in Figure 1b [2.291(2) Å].^9^ The Si–S bond distances of **2** [2.1237(18)–2.1387(18) Å] are similar to that (2.173 Å) in **2-Me** and that [2.1381(6) Å] in four-coordinate silicon bis­(dithiolene), Si­[S_2_(*o*-C_6_H_4_)]_2_ (Figure 2 **A**).^17^ The two four-coordinate silicon atoms in **2** adopt a tetrahedral geometry (τ_4_ = 0.98 and 0.99, respectively).^26^ The olefinic C–C bonds [1.346(6)–1.353(6) Å] and C–S bonds [1.749(5)–1.764(5) Å] in the dithiolene (i.e., C_2_S_2_) units of **2** are comparable with those [d_C=C_ = 1.342(4) Å; d_C–S_ = 1.727(3) Å] in a silicon tris­(dithiolate) dianion (Figure 2 **C**).^20^ Consequently, the silicon atoms in **2** are in the formal oxidation state of +3.

**Figure 3:**
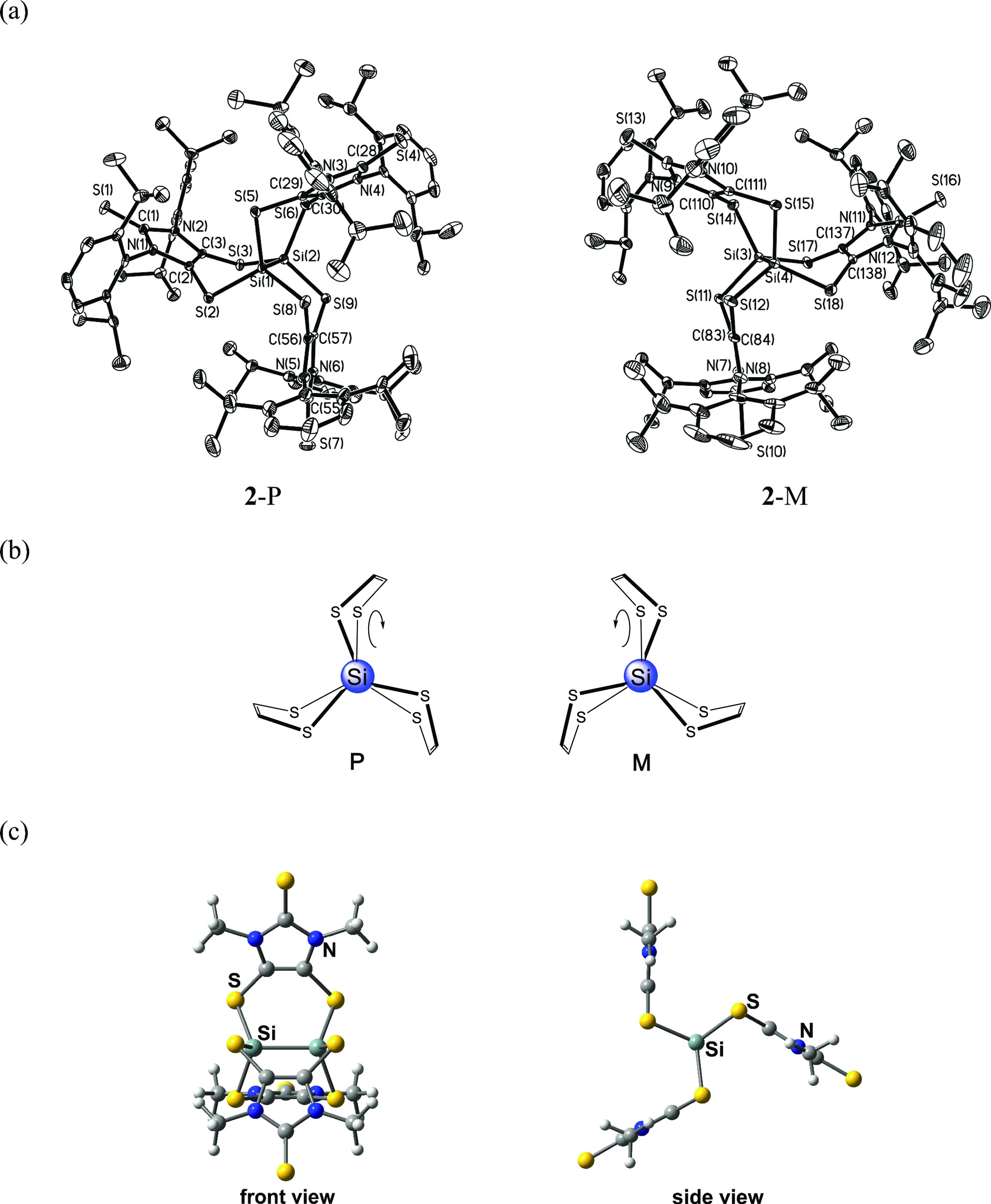
(a) Molecular structures of **2** (P- and M-helical enantiomers). Thermal ellipsoids represent 30% probability. Hydrogen atoms have been omitted for clarity. Selected bond distances (Å) and angles (deg): for **2**-P, Si(1)–Si(2) 2.2936(19); Si(1)–S(2) 2.1300(17); C(2)–S(2) 1.759(5); C(2)–C(3) 1.353(6); Si(2)–Si(1)–S(2) 108.18(7); S(2)–Si(1)–S(5) 110.84(7); S(2)–Si(1)–S(8) 111.59(7); S(5)–Si(1)–S(8) 109.84(7). (b) Schematic representation of the “hexathiadisila[4.4.4]­propellene” core of **2** (P- and M-helical enantiomers) (viewed down the Si–Si bridgehead axis). (c) **2-Me** model in *C*_3*h*_ symmetry.

The HOMO and HOMO–1 of **2-Me** are dithiolene ligand-based (Figure 4). While the LUMO of **2-Me** is dominated by Si–Si σ*-antibonding character, the HOMO–6 involves Si–Si σ-bonding character (Figure 4). Natural bond orbital (NBO) analysis shows that each silicon atom in **2-Me** bears a positive charge of +0.66. The silicon–silicon bond in **2-Me** (Wiberg bond index = 0.811) has 27.4% s-, 72.1% p-, and 0.5% d-character. The LUMO of **2-Me** (ca. −2.48 eV) reveals that **2** may act as an electron receiver in single-electron transfer processes and be thus utilized in redox-induced small-molecule activation. The TD-DFT computations (CAM-B3LYP/6-311G**, SMD, toluene) of **2-Me** reveals that the HOMO → LUMO transition is optically dark (f ≈ 0); the intense UV absorption () originates from higher-energy allowed excitations.

**Figure 4:**
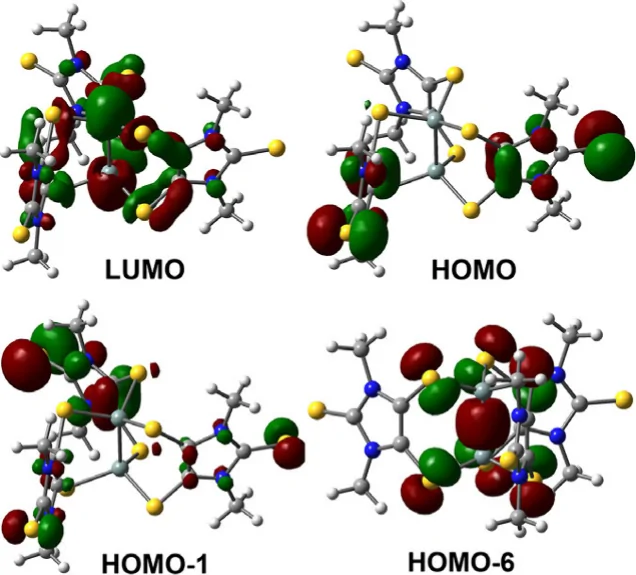
Selected molecular orbitals of the **2-Me** model (silicon, light cyan; sulfur, yellow; carbon, gray; nitrogen, blue; hydrogen, white).

The first hexathiadisila[4.4.4]­propellene with a central silicon–silicon bond (**2**) was obtained via reaction of an imidazole-based dithiolate dimer (**1**) with Cl_3_Si–SiCl_3_ in toluene. DFT computations suggest that the helical chirality of **2** is induced by the steric bulk of the dithiolene ligands. The potential utility of **2** in small-molecule activation is being investigated in this laboratory.

## Supplementary Material





## Notes

Cartesian coordinates for the calculated structures (XYZ)
Syntheses, computations, and X-ray crystal determination (PDF)
