# Supplementary material for: Flash Communication: A Dithiolene-Based Hexathiadisila[4.4.4]propellene
Source: Organometallics. 2026 Aug 12;45(16):1874–8. doi: 10.1021/acs.organomet.6c00227 (PMC13509123; doi:10.1021/acs.organomet.6c00227)
Supplement: Supplementary file 1 [file om6c00227_si_002.pdf]

Supplementary Information

Flash Communication: A Dithiolene-Based Hexathiadisila[4.4.4]propellene

John C. Johnson, Yuzhong Wang, Claire Buck, Pingrong Wei, Mitchell E. Lahm, Henry F. Schaefer, III, and Gregory H. Robinson*

Department of Chemistry and the Center for Computational Chemistry, The University of Georgia, Athens, Georgia 30602-2556 (USA)

*To whom correspondence should be addressed.

Email: robinson@uga.edu

Table of Contents **Page**

Synthesis and spectral data S2-S6

Computational data S7

X-Ray Crystallography Data S8-S19

SUPPORTING INFORMATION of SYNTHESES

Materials and Methods

General.

All reactions were performed under purified argon using Schlenk techniques and an inert atmosphere drybox (M-Braun LabMaster 130). Chemicals were purchased from commercial sources and used as received. Solvents were dried and distilled under argon from Na/benzophenone prior to use. ^{1}H, $^{13}C\{^{1}H\}$, and $^{29}Si\{^{1}H\}$ NMR spectra were recorded on a Bruker Avance III HD 400 MHz spectrometer. The $^{29}Si\{^{1}H\}$ NMR spectra were referenced to TMS as an external standard. X-ray intensity data for **2** (CCDC# 2561849) was collected at 135K on a Bruker D8 Quest PHOTON 100 CMOS X-ray diffractometer system with Incoatec Microfocus Source (IμS) monochromated Mo Kα radiation (λ = 0.71073 Å, sealed tube) using phi and omega-scan technique.

Compound 2: Hexachlorodisilane (0.024 g, 0.088 mmol) in Toluene (1 mL) was quickly added to a stirred solution of **1** (0.150 g, 0.088 mmol) in Toluene (10 mL). The reaction mixture was protected from light and stirred for 24h at ambient temperature. The volatiles were removed in vacuo after filtration. The residue was subsequently washed with 10 mL of hexanes, affording pale yellow crystalline solid of **2** (0.057 g, in 65% yield). X-ray quality colorless crystals of **2** were obtained by recrystallization in toluene/hexanes solvent mixture. Mp: > 275 °C gradually melted and decomposed. ^{1}H NMR (400.22 MHz, C_6D_6): δ 1.24 [d, 36H, $CH(CH_3)_2$], 1.47 [d, 36H, $CH(CH_3)_2$], 2.83 [bs, 12H, $CH(CH_3)_2$], 7.12 (d, 12H, Ar-*H*), 7.25 (m, 6H, Ar-*H*). $^{13}C\{^{1}H\}$ NMR (100.65 MHz, C_6D_6): δ 24.8, 25.2 [$CH(CH_3)_2$], 30.2 [$CH(CH_3)_2$], 118.1 (S−*C*=*C*−S), 124.9, 131.3, 132.2, 147.9 (Ar-*C*), 171.1 [N(*C*=S)N]. $^{29}Si\{^{1}H\}$ NMR (79.51 MHz, PhBr-d_5): δ 14.7. Crystal data for **2**: $C_{169}H_{212}N_{12}S_{18}Si_4$, fw = 3100.93 g/mol, monoclinic, $P2_1/n$, a = 26.6485(19) Å, b = 25.5802(18) Å, c = 28.402(2) Å, β = 111.432(2)°, V = 18022(2) Å^3, Z = 4, R1 = 0.0818 for 16725 data (I>2σ(I)), wR2 = 0.1661 (all data).

Compound **2**

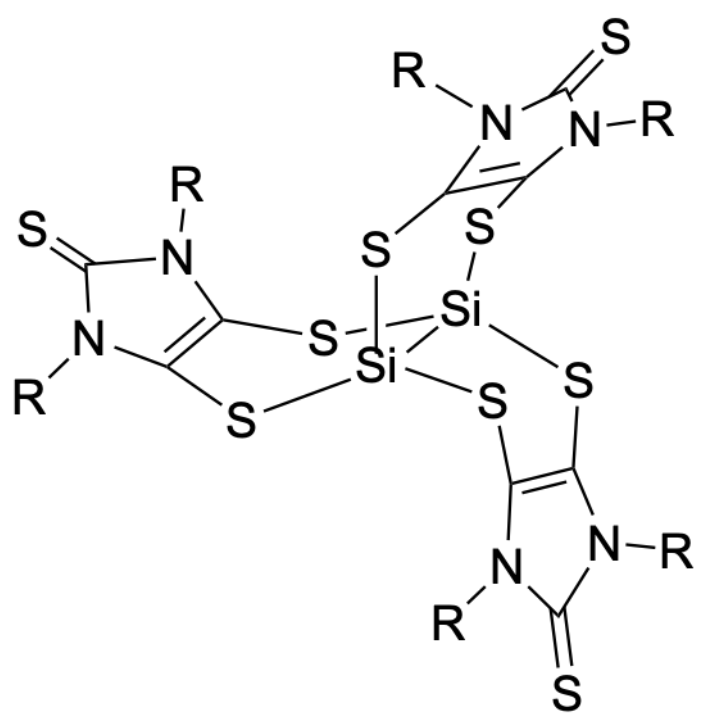

(R = 2,6-diisopropylphenyl)

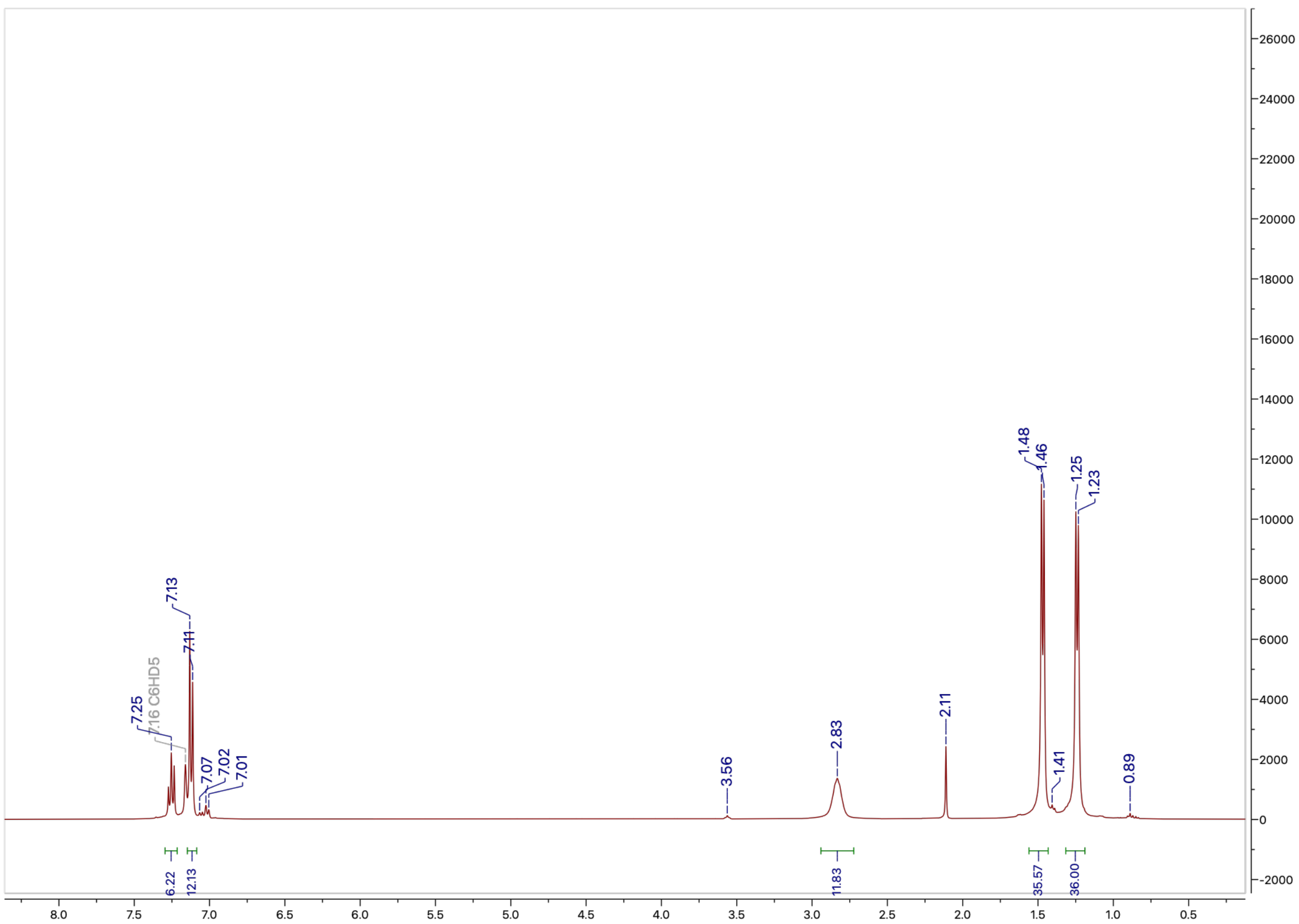

Figure S1. ^{1}H NMR spectrum of **2** in C_6D_6 (toluene: 2.11 ppm, 7.01-7.07 ppm; THF: 1.41 and 3.56 ppm; hexane: 0.89 ppm).

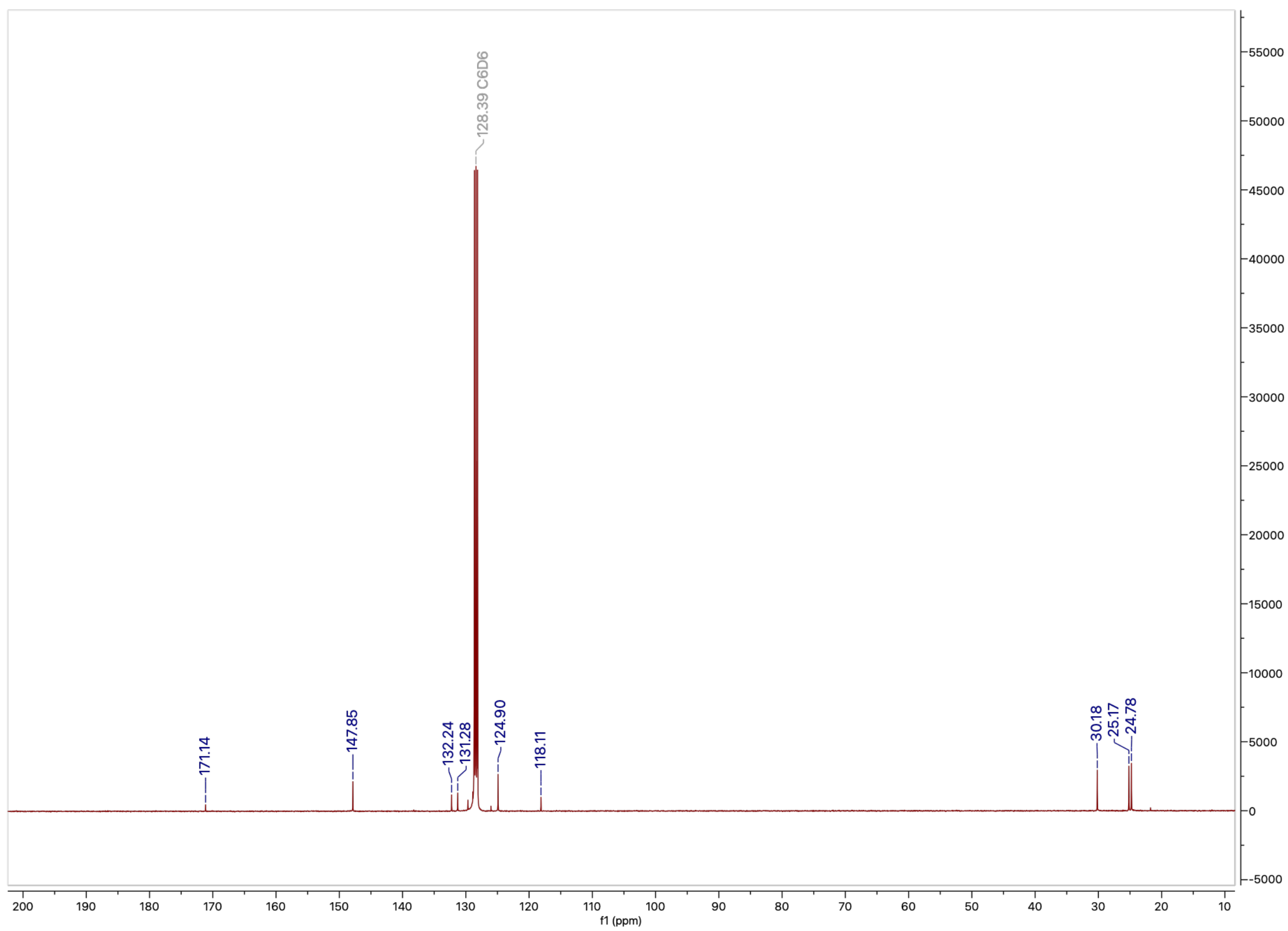

Figure S2. $^{13}C\{^1H\}$ spectrum of **2** in C_6D_6.

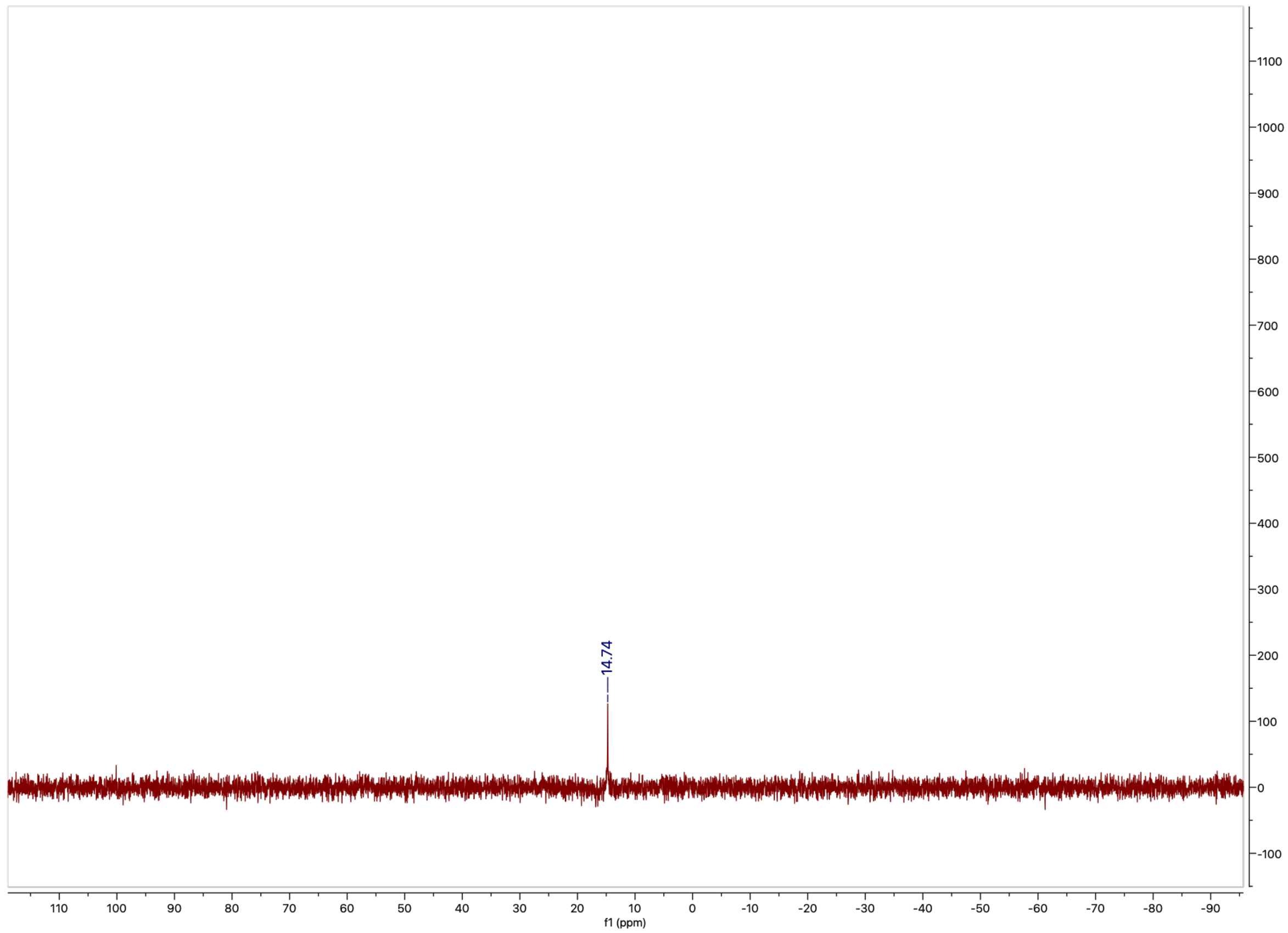

Figure S3. $^{29}Si\{^1H\}$ NMR spectrum of **2** in PhBr-d_5.

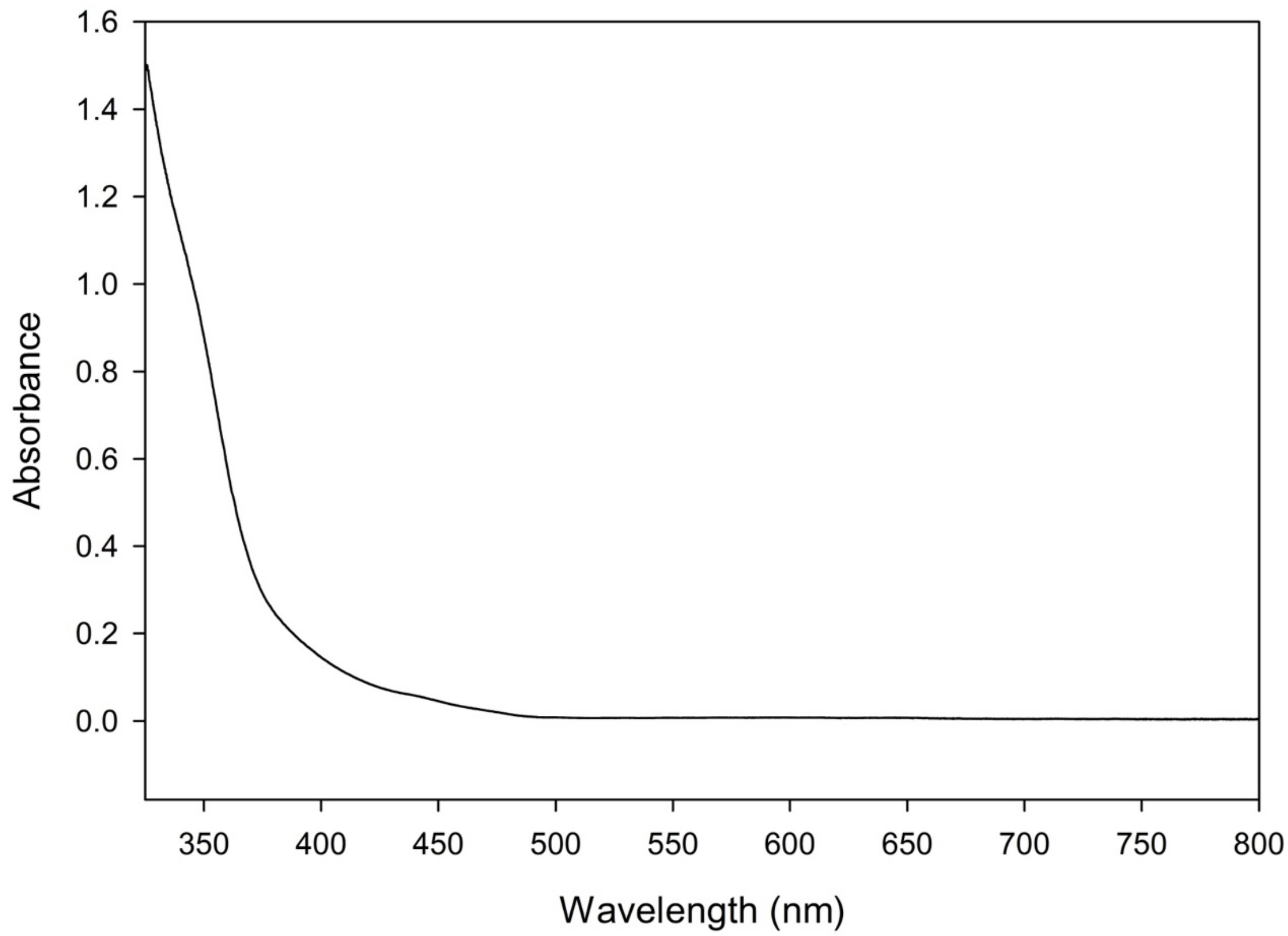

Figure S4. UV-vis spectrum of **2** in toluene.

SUPPORTING INFORMATION of COMPUTATIONS

All computations employed the Gaussian 16 (Revision C.01) program:

Gaussian 16, Revision C.01,
Frisch, M. J.; Trucks, G. W.; Schlegel, H. B.; Scuseria, G. E.;
Robb, M. A.; Cheeseman, J. R.; Scalmani, G.; Barone, V.;
Petersson, G. A.; Nakatsuji, H.; Li, X.; Caricato, M.; Marenich, A. V.;
Bloino, J.; Janesko, B. G.; Gomperts, R.; Mennucci, B.; Hratchian, H. P.;
Ortiz, J. V.; Izmaylov, A. F.; Sonnenberg, J. L.; Williams-Young, D.;
Ding, F.; Lipparini, F.; Egidi, F.; Goings, J.; Peng, B.; Petrone, A.;
Henderson, T.; Ranasinghe, D.; Zakrzewski, V. G.; Gao, J.; Rega, N.;
Zheng, G.; Liang, W.; Hada, M.; Ehara, M.; Toyota, K.; Fukuda, R.;
Hasegawa, J.; Ishida, M.; Nakajima, T.; Honda, Y.; Kitao, O.; Nakai, H.
Vreven, T.; Throssell, K.; Montgomery, Jr., J. A.; Peralta, J. E.;
Ogliaro, F.; Bearpark, M. J.; Heyd, J. J.; Brothers, E. N.; Kudin, K. N.;
Staroverov, V. N.; Keith, T. A.; Kobayashi, R.; Normand, J.;
Raghavachari, K.; Rendell, A. P.; Burant, J. C.; Iyengar, S. S.;
Tomasi, J.; Cossi, M.; Millam, J. M.; Klene, M.; Adamo, C.; Cammi, R.;
Ochterski, J. W.; Martin, R. L.; Morokuma, K.; Farkas, O.;
Foresman, J. B. and Fox, D. J. Gaussian, Inc., Wallingford CT, 2019.

SUPPORTING INFORMATION of X-RAY

Compound **2**

Table S1. Sample and crystal data for **2**.

**Identification code**	**2**	
**Chemical formula**	$C_{169}H_{212}N_{12}S_{18}Si_4$	
**Formula weight**	3100.93 g/mol	
**Temperature**	135(2) K	
**Wavelength**	0.71073 Å	
**Crystal size**	0.080 x 0.140 x 0.180 mm	
**Crystal system**	monoclinic	
**Space group**	$P2_1/n$ (No. 14)	
**Unit cell dimensions**	a = 26.6485(19) Å	α = 90°
	b = 25.5802(18) Å	β = 111.432(2)°
	c = 28.402(2) Å	γ = 90°
**Volume**	18022(2) $Å^3$	
**Z**	4	
**Density (calculated)**	1.143 g/cm^3	
**Absorption coefficient**	0.291 mm^{-1}	
**F(000)**	6616	

Table S2. Data collection and structure refinement for **2**.

**Theta range for data collection**	1.73 to 25.48°
**Index ranges**	-32<=h<=32, -30<=k<=30, -34<=l<=34
**Reflections collected**	455910
**Independent reflections**	33358 [R(int) = 0.3490]
**Max. and min. transmission**	0.7452 and 0.6529
**Structure solution technique**	direct methods
**Structure solution program**	SHELXT 2018/2 (Sheldrick, 2018)
**Refinement method**	Full-matrix least-squares on F^2
**Refinement program**	SHELXL-2019/2 (Sheldrick, 2019)
**Function minimized**	$\Sigma\ w(F_o^2 - F_c^2)^2$
**Data / restraints / parameters**	33358 / 0 / 1828
**Goodness-of-fit on F^2**	1.002
**Δ/σ_{max}**	0.001
**Final R indices**	16725 data; I>2σ(I) R1 = 0.0818, wR2 = 0.1316
	all data R1 = 0.1928, wR2 = 0.1661
**Weighting scheme**	$w=1/[\sigma^2(F_o^2)+(0.0483P)^2+28.8209P]$<br>where $P=(F_o^2+2F_c^2)/3$
**Largest diff. peak and hole**	0.380 and -0.381 eÅ^{-3}
**R.M.S. deviation from mean**	0.076 eÅ^{-3}

Table S3. Bond lengths (Å) for **2.**

Si1-S5	2.1271(18)	Si1-S8	2.1325(18)
Si1-S2	2.1300(17)	Si1-Si2	2.2936(19)
Si2-S6	2.1320(17)	Si2-S3	2.1352(17)
Si2-S9	2.1354(17)	Si3-S11	2.1237(18)
Si3-S14	2.1279(18)	Si3-S17	2.1349(18)
Si3-Si4	2.2958(19)	Si4-S18	2.1256(18)
Si4-S15	2.1268(18)	Si4-S12	2.1387(18)
S1-C1	1.653(5)	S2-C2	1.759(5)
S3-C3	1.752(5)	S4-C28	1.663(5)
S5-C29	1.755(5)	S6-C30	1.750(5)
S7-C55	1.663(5)	S8-C56	1.756(5)
S9-C57	1.764(5)	S10-C82	1.664(5)
S11-C83	1.754(5)	S12-C84	1.758(5)
S13-C109	1.649(5)	S14-C110	1.754(5)
S15-C111	1.755(5)	S16-C136	1.658(5)
S17-C137	1.759(5)	S18-C138	1.749(5)
N1-C1	1.382(6)	N1-C2	1.401(5)
N1-C16	1.452(6)	N2-C1	1.377(6)
N2-C3	1.399(5)	N2-C4	1.457(6)
N3-C28	1.368(6)	N3-C29	1.398(6)
N3-C43	1.450(6)	N4-C28	1.374(6)
N4-C30	1.403(5)	N4-C31	1.436(6)
N5-C55	1.368(6)	N5-C56	1.392(6)
N5-C70	1.444(6)	N6-C55	1.372(6)
N6-C57	1.395(5)	N6-C58	1.446(6)
N7-C82	1.377(6)	N7-C83	1.396(5)
N7-C97	1.444(6)	N8-C82	1.369(6)

N8-C84	1.405(6)	N8-C85	1.455(6)
N9-C109	1.376(6)	N9-C110	1.397(6)
N9-C124	1.440(6)	N10-C109	1.379(6)
N10-C111	1.397(6)	N10-C112	1.457(6)
N11-C136	1.377(6)	N11-C137	1.404(6)
N11-C151	1.445(6)	N12-C136	1.370(6)
N12-C138	1.395(6)	N12-C139	1.455(6)
C2-C3	1.353(6)	C4-C9	1.394(6)
C4-C5	1.389(6)	C5-C6	1.394(6)
C5-C13	1.516(7)	C6-C7	1.382(7)
C7-C8	1.372(7)	C8-C9	1.396(7)
C9-C10	1.518(7)	C10-C12	1.531(7)
C10-C11	1.530(7)	C13-C14	1.520(7)
C13-C15	1.528(6)	C16-C21	1.395(6)
C16-C17	1.399(6)	C17-C18	1.385(7)
C17-C25	1.523(7)	C18-C19	1.372(7)
C19-C20	1.373(7)	C20-C21	1.396(7)
C21-C22	1.514(7)	C22-C24	1.535(7)
C22-C23	1.530(7)	C25-C26	1.521(7)
C25-C27	1.526(7)	C29-C30	1.352(6)
C31-C36	1.388(7)	C31-C32	1.401(7)
C32-C33	1.388(7)	C32-C40	1.519(7)
C33-C34	1.368(8)	C34-C35	1.381(8)
C35-C36	1.397(7)	C36-C37	1.514(7)
C37-C39	1.528(8)	C37-C38	1.523(7)
C40-C41	1.530(7)	C40-C42	1.538(7)
C43-C48	1.390(8)	C43-C44	1.394(7)
C44-C45	1.400(8)	C44-C52	1.514(8)
C45-C46	1.363(10)	C46-C47	1.375(10)

C47-C48	1.383(8)	C48-C49	1.506(9)
C49-C50	1.507(8)	C49-C51	1.530(8)
C52-C54	1.524(8)	C52-C53	1.541(8)
C56-C57	1.350(6)	C58-C63	1.393(7)
C58-C59	1.391(7)	C59-C60	1.392(7)
C59-C67	1.504(7)	C60-C61	1.371(7)
C61-C62	1.382(8)	C62-C63	1.391(7)
C63-C64	1.521(7)	C64-C66	1.534(7)
C64-C65	1.534(7)	C67-C69	1.534(7)
C67-C68	1.528(7)	C70-C75	1.394(7)
C70-C71	1.400(7)	C71-C72	1.382(7)
C71-C79	1.517(7)	C72-C73	1.379(8)
C73-C74	1.370(8)	C74-C75	1.390(8)
C75-C76	1.509(8)	C76-C77	1.521(7)
C76-C78	1.555(8)	C79-C81	1.508(8)
C79-C80	1.534(7)	C83-C84	1.346(6)
C85-C90	1.380(8)	C85-C86	1.401(8)
C86-C87	1.394(8)	C86-C94	1.514(9)
C87-C88	1.361(10)	C88-C89	1.380(10)
C89-C90	1.397(8)	C90-C91	1.504(8)
C91-C92	1.531(7)	C91-C93	1.540(8)
C94-C95	1.521(8)	C94-C96	1.528(9)
C97-C102	1.391(6)	C97-C98	1.385(6)
C98-C99	1.399(7)	C98-C106	1.520(7)
C99-C100	1.372(7)	C100-C101	1.360(7)
C101-C102	1.386(7)	C102-C103	1.524(7)
C103-C104	1.524(7)	C103-C105	1.510(7)
C106-C107	1.512(7)	C106-C108	1.517(7)
C110-C111	1.351(6)	C112-C117	1.382(7)

C112-C113	1.405(7)	C113-C114	1.396(8)
C113-C121	1.516(7)	C114-C115	1.366(9)
C115-C116	1.352(9)	C116-C117	1.420(8)
C117-C118	1.485(8)	C118-C119	1.526(9)
C118-C120	1.551(9)	C121-C122	1.516(8)
C121-C123	1.531(7)	C124-C125	1.390(7)
C124-C129	1.388(7)	C125-C126	1.389(7)
C125-C133	1.508(7)	C126-C127	1.378(8)
C127-C128	1.376(8)	C128-C129	1.401(7)
C129-C130	1.505(7)	C130-C131	1.534(8)
C130-C132	1.523(7)	C133-C134	1.515(8)
C133-C135	1.515(8)	C137-C138	1.352(6)
C139-C144	1.389(8)	C139-C140	1.399(8)
C140-C141	1.388(8)	C140-C148	1.495(8)
C141-C142	1.370(10)	C142-C143	1.380(10)
C143-C144	1.412(9)	C144-C145	1.491(8)
C145-C147	1.523(8)	C145-C146	1.526(9)
C148-C150	1.532(8)	C148-C149	1.541(7)
C151-C152	1.389(7)	C151-C156	1.393(7)
C152-C153	1.396(7)	C152-C160	1.517(7)
C153-C154	1.368(8)	C154-C155	1.371(8)
C155-C156	1.387(7)	C156-C157	1.520(8)
C157-C159	1.538(7)	C157-C158	1.539(7)
C160-C161	1.524(8)	C160-C162	1.528(7)
C1A-C1B	1.398(10)	C1A-C1F	1.408(11)
C1A-C1G	1.477(11)	C1B-C1C	1.375(10)
C1C-C1D	1.352(9)	C1D-C1E	1.327(10)
C1E-C1F	1.379(11)		

Table S4. Bond angles (°) for **2.**

S5-Si1-S8	109.84(7)	S5-Si1-S2	110.84(7)
S8-Si1-S2	111.59(7)	S5-Si1-Si2	108.00(7)
S8-Si1-Si2	108.27(7)	S2-Si1-Si2	108.18(7)
S6-Si2-S3	108.89(7)	S6-Si2-S9	108.98(7)
S3-Si2-S9	110.43(7)	S6-Si2-Si1	109.64(7)
S3-Si2-Si1	109.61(7)	S9-Si2-Si1	109.27(7)
S11-Si3-S14	107.18(7)	S11-Si3-S17	110.73(7)
S14-Si3-S17	110.47(8)	S11-Si3-Si4	108.27(7)
S14-Si3-Si4	110.29(7)	S17-Si3-Si4	109.85(7)
S18-Si4-S15	110.12(8)	S18-Si4-S12	109.75(8)
S15-Si4-S12	111.90(8)	S18-Si4-Si3	108.44(8)
S15-Si4-Si3	107.22(7)	S12-Si4-Si3	109.32(8)
C2-S2-Si1	95.75(16)	C3-S3-Si2	100.51(16)
C29-S5-Si1	95.08(16)	C30-S6-Si2	99.27(16)
C56-S8-Si1	95.83(17)	C57-S9-Si2	100.00(15)
C83-S11-Si3	99.43(16)	C84-S12-Si4	95.27(16)
C110-S14-Si3	99.16(16)	C111-S15-Si4	96.27(16)
C137-S17-Si3	100.87(16)	C138-S18-Si4	97.24(16)
C1-N1-C2	110.3(4)	C1-N1-C16	123.2(4)
C2-N1-C16	125.3(4)	C1-N2-C3	111.3(4)
C1-N2-C4	124.5(4)	C3-N2-C4	124.0(4)
C28-N3-C29	110.2(4)	C28-N3-C43	124.8(4)
C29-N3-C43	123.7(4)	C28-N4-C30	110.3(4)
C28-N4-C31	124.5(4)	C30-N4-C31	124.8(4)
C55-N5-C56	110.1(4)	C55-N5-C70	124.2(4)
C56-N5-C70	123.9(4)	C55-N6-C57	110.2(4)
C55-N6-C58	125.0(4)	C57-N6-C58	124.7(4)

C82-N7-C83	109.9(4)	C82-N7-C97	125.5(4)
C83-N7-C97	124.3(4)	C82-N8-C84	109.8(4)
C82-N8-C85	122.2(4)	C84-N8-C85	126.7(4)
C109-N9-C110	111.0(4)	C109-N9-C124	122.5(4)
C110-N9-C124	126.2(4)	C109-N10-C111	110.7(4)
C109-N10-C112	124.5(4)	C111-N10-C112	122.4(4)
C136-N11-C137	110.0(4)	C136-N11-C151	124.9(4)
C137-N11-C151	125.2(4)	C136-N12-C138	110.6(4)
C136-N12-C139	124.0(4)	C138-N12-C139	124.2(4)
N2-C1-N1	104.1(4)	N2-C1-S1	128.2(4)
N1-C1-S1	127.7(4)	C3-C2-N1	107.7(4)
C3-C2-S2	132.4(4)	N1-C2-S2	119.8(3)
C2-C3-N2	106.4(4)	C2-C3-S3	132.8(4)
N2-C3-S3	120.4(3)	C9-C4-C5	123.8(4)
C9-C4-N2	118.1(4)	C5-C4-N2	118.1(4)
C6-C5-C4	116.5(5)	C6-C5-C13	120.3(4)
C4-C5-C13	123.1(4)	C7-C6-C5	121.8(5)
C6-C7-C8	119.6(5)	C7-C8-C9	121.6(5)
C4-C9-C8	116.7(5)	C4-C9-C10	123.2(4)
C8-C9-C10	120.1(5)	C9-C10-C12	110.9(4)
C9-C10-C11	111.7(4)	C12-C10-C11	110.2(4)
C5-C13-C14	111.5(4)	C5-C13-C15	113.5(4)
C14-C13-C15	109.6(4)	C21-C16-C17	123.7(4)
C21-C16-N1	118.9(4)	C17-C16-N1	117.4(4)
C18-C17-C16	116.3(5)	C18-C17-C25	119.9(4)
C16-C17-C25	123.6(4)	C19-C18-C17	121.7(5)
C20-C19-C18	120.6(5)	C19-C20-C21	120.9(5)
C16-C21-C20	116.6(5)	C16-C21-C22	123.4(5)
C20-C21-C22	120.0(5)	C21-C22-C24	111.7(5)
C21-C22-C23	110.0(4)	C24-C22-C23	110.5(4)

C17-C25-C26	109.1(4)	C17-C25-C27	112.3(4)
C26-C25-C27	111.2(4)	N4-C28-N3	105.1(4)
N4-C28-S4	127.4(4)	N3-C28-S4	127.5(4)
C30-C29-N3	107.5(4)	C30-C29-S5	132.2(4)
N3-C29-S5	120.2(4)	C29-C30-N4	106.7(4)
C29-C30-S6	132.7(4)	N4-C30-S6	120.2(4)
C36-C31-C32	123.4(5)	C36-C31-N4	119.2(4)
C32-C31-N4	117.4(4)	C33-C32-C31	116.6(5)
C33-C32-C40	119.9(5)	C31-C32-C40	123.4(5)
C34-C33-C32	122.0(6)	C33-C34-C35	119.8(6)
C34-C35-C36	121.4(6)	C31-C36-C35	116.8(5)
C31-C36-C37	122.0(5)	C35-C36-C37	121.1(5)
C36-C37-C39	110.9(5)	C36-C37-C38	113.0(5)
C39-C37-C38	109.5(5)	C32-C40-C41	111.1(5)
C32-C40-C42	112.6(5)	C41-C40-C42	110.4(4)
C48-C43-N3	116.8(5)	C48-C43-C44	124.4(5)
N3-C43-C44	118.7(5)	C43-C44-C45	115.8(6)
C43-C44-C52	122.6(5)	C45-C44-C52	121.6(6)
C46-C45-C44	121.5(7)	C45-C46-C47	120.4(7)
C48-C47-C46	121.7(7)	C43-C48-C47	116.2(7)
C43-C48-C49	122.9(5)	C47-C48-C49	120.8(6)
C48-C49-C50	110.4(6)	C48-C49-C51	115.1(6)
C50-C49-C51	110.8(5)	C54-C52-C44	111.0(6)
C54-C52-C53	110.6(5)	C44-C52-C53	111.0(5)
N6-C55-N5	105.1(4)	N6-C55-S7	127.6(4)
N5-C55-S7	127.3(4)	C57-C56-N5	107.5(4)
C57-C56-S8	132.3(4)	N5-C56-S8	120.2(4)
C56-C57-N6	106.9(4)	C56-C57-S9	132.2(4)
N6-C57-S9	120.3(3)	C63-C58-C59	123.6(5)
C63-C58-N6	118.1(4)	C59-C58-N6	118.2(5)

C60-C59-C58	116.6(5)	C60-C59-C67	120.1(5)
C58-C59-C67	123.3(5)	C61-C60-C59	121.1(5)
C60-C61-C62	121.1(5)	C61-C62-C63	120.1(5)
C62-C63-C58	117.5(5)	C62-C63-C64	121.3(5)
C58-C63-C64	121.2(5)	C66-C64-C63	111.6(4)
C66-C64-C65	109.4(5)	C63-C64-C65	113.4(5)
C59-C67-C69	112.7(5)	C59-C67-C68	111.4(5)
C69-C67-C68	109.7(5)	C75-C70-C71	123.6(5)
C75-C70-N5	119.3(5)	C71-C70-N5	117.2(5)
C72-C71-C70	117.1(5)	C72-C71-C79	119.3(5)
C70-C71-C79	123.5(5)	C73-C72-C71	120.6(6)
C74-C73-C72	121.0(6)	C73-C74-C75	121.2(6)
C70-C75-C74	116.5(5)	C70-C75-C76	122.4(5)
C74-C75-C76	121.1(5)	C77-C76-C75	110.7(5)
C77-C76-C78	111.5(5)	C75-C76-C78	110.0(5)
C81-C79-C71	114.3(5)	C81-C79-C80	110.9(5)
C71-C79-C80	109.1(5)	N7-C82-N8	105.3(4)
N7-C82-S10	126.8(4)	N8-C82-S10	127.9(4)
C84-C83-N7	107.4(4)	C84-C83-S11	132.6(4)
N7-C83-S11	119.6(3)	C83-C84-N8	107.4(4)
C83-C84-S12	131.6(4)	N8-C84-S12	121.0(4)
C90-C85-C86	125.0(5)	C90-C85-N8	118.3(5)
C86-C85-N8	116.7(5)	C87-C86-C85	115.2(6)
C87-C86-C94	121.0(6)	C85-C86-C94	123.8(5)
C88-C87-C86	122.1(7)	C89-C88-C87	120.6(6)
C88-C89-C90	120.8(7)	C85-C90-C89	116.3(6)
C85-C90-C91	124.1(5)	C89-C90-C91	119.6(6)
C90-C91-C92	112.8(5)	C90-C91-C93	111.5(5)
C92-C91-C93	109.7(5)	C86-C94-C95	112.6(6)
C86-C94-C96	111.6(6)	C95-C94-C96	110.2(6)

C102-C97-C98	123.6(5)	C102-C97-N7	118.2(4)
C98-C97-N7	118.1(4)	C97-C98-C99	116.7(5)
C97-C98-C106	123.0(4)	C99-C98-C106	120.2(4)
C100-C99-C98	120.5(5)	C99-C100-C101	121.1(5)
C102-C101-C100	121.2(5)	C101-C102-C97	116.7(5)
C101-C102-C103	121.3(5)	C97-C102-C103	121.9(5)
C104-C103-C105	109.2(5)	C104-C103-C102	113.1(5)
C105-C103-C102	111.8(5)	C107-C106-C108	110.9(4)
C107-C106-C98	111.5(4)	C108-C106-C98	111.7(4)
N10-C109-N9	103.9(4)	N10-C109-S13	127.3(4)
N9-C109-S13	128.8(4)	C111-C110-N9	106.8(4)
C111-C110-S14	131.6(4)	N9-C110-S14	120.3(4)
C110-C111-N10	107.3(4)	C110-C111-S15	133.1(4)
N10-C111-S15	119.6(4)	C117-C112-C113	124.7(5)
C117-C112-N10	118.8(5)	C113-C112-N10	116.6(5)
C112-C113-C114	115.9(6)	C112-C113-C121	124.1(5)
C114-C113-C121	119.9(5)	C115-C114-C113	120.7(6)
C114-C115-C116	122.3(7)	C115-C116-C117	120.6(7)
C112-C117-C116	115.8(6)	C112-C117-C118	123.0(6)
C116-C117-C118	121.2(6)	C117-C118-C119	111.4(6)
C117-C118-C120	111.1(6)	C119-C118-C120	111.0(6)
C113-C121-C122	114.0(5)	C113-C121-C123	109.7(5)
C122-C121-C123	110.7(5)	C125-C124-C129	124.6(5)
C125-C124-N9	117.1(4)	C129-C124-N9	118.3(4)
C124-C125-C126	116.8(5)	C124-C125-C133	123.3(5)
C126-C125-C133	119.8(5)	C127-C126-C125	120.6(5)
C126-C127-C128	121.0(6)	C127-C128-C129	121.0(5)
C124-C129-C128	116.0(5)	C124-C129-C130	122.3(5)
C128-C129-C130	121.7(5)	C131-C130-C129	110.9(5)
C131-C130-C132	110.2(5)	C129-C130-C132	113.4(5)

C134-C133-C135	111.0(5)	C134-C133-C125	111.5(5)
C135-C133-C125	112.1(5)	N11-C136-N12	105.0(4)
N11-C136-S16	127.0(4)	N12-C136-S16	128.0(4)
C138-C137-N11	107.2(4)	C138-C137-S17	132.7(4)
N11-C137-S17	119.7(3)	C137-C138-N12	107.2(4)
C137-C138-S18	133.0(4)	N12-C138-S18	119.9(4)
C144-C139-C140	124.4(5)	C144-C139-N12	116.7(5)
C140-C139-N12	118.9(5)	C139-C140-C141	116.4(6)
C139-C140-C148	122.8(5)	C141-C140-C148	120.8(6)
C142-C141-C140	121.8(7)	C141-C142-C143	120.3(7)
C142-C143-C144	121.1(7)	C139-C144-C143	116.0(6)
C139-C144-C145	123.7(5)	C143-C144-C145	120.3(6)
C144-C145-C147	111.8(6)	C144-C145-C146	111.9(6)
C147-C145-C146	110.8(6)	C140-C148-C150	111.6(5)
C140-C148-C149	112.7(5)	C150-C148-C149	109.9(5)
C152-C151-C156	123.8(5)	C152-C151-N11	118.0(5)
C156-C151-N11	118.2(5)	C153-C152-C151	116.7(5)
C153-C152-C160	120.9(5)	C151-C152-C160	122.4(5)
C154-C153-C152	120.7(6)	C155-C154-C153	121.2(6)
C154-C155-C156	120.9(6)	C151-C156-C155	116.7(5)
C151-C156-C157	123.1(5)	C155-C156-C157	120.2(5)
C156-C157-C159	111.4(5)	C156-C157-C158	111.8(5)
C159-C157-C158	109.2(5)	C161-C160-C152	110.8(5)
C161-C160-C162	110.5(5)	C152-C160-C162	112.8(5)
C1B-C1A-C1F	116.3(8)	C1B-C1A-C1G	120.6(10)
C1F-C1A-C1G	123.1(10)	C1C-C1B-C1A	119.9(8)
C1D-C1C-C1B	123.4(8)	C1E-C1D-C1C	117.0(9)
C1D-C1E-C1F	123.8(9)	C1E-C1F-C1A	119.7(8)
